# Shaping Rolling Circle Amplification Products into DNA Nanoparticles by Incorporation of Modified Nucleotides and Their Application to In Vitro and In Vivo Delivery of a Photosensitizer

**DOI:** 10.3390/molecules23071833

**Published:** 2018-07-23

**Authors:** Kyoung-Ran Kim, Pascal Röthlisberger, Seong Jae Kang, Kihwan Nam, Sangyoup Lee, Marcel Hollenstein, Dae-Ro Ahn

**Affiliations:** 1 Center for Theragnosis, Biomedical Research Institute, Korea Institute of Science and Technology (KIST), Hwarangno 14-gil 5, Seongbuk-gu, Seoul 02792, Korea; krkim830@kist.re.kr (K.-R.K.); sjkang@kist.re.kr (S.J.K.); 2 Department of Structural Biology and Chemistry, Laboratory for Bioorganic Chemistry of Nucleic Acids, Institut Pasteur, CNRS UMR3523, 28, rue du Docteur Roux, 75724 Paris CEDEX 15, France; pascal.rothlisberger@pasteur.fr (P.R.); marcel.hollenstein@pasteur.fr (M.H.); 3 Center for Bionics, Biomedical Research Institute, Korea Institute of Science and Technology (KIST), Hwarangno 14-gil 5, Seongbuk-gu, Seoul 02792, Korea; kihwannam@gmail.com (K.N.); sangyoup@kist.re.kr (S.L.); 4 Division of Biomedical Science and Technology, KIST School, Korea University of Science and Technology (UST), Hwarangno 14-gil 5, Seongbuk-gu, Seoul 02792, Korea

**Keywords:** rolling circle amplification, DNA nanoparticles, modified nucleotides, photosensitizer, photodynamic therapy

## Abstract

Rolling circle amplification (RCA) is a robust way to generate DNA constructs, which are promising materials for biomedical applications including drug delivery because of their high biocompatibility. To be employed as a drug delivery platform, however, the DNA materials produced by RCA need to be shaped into nanoparticles that display both high cellular uptake efficiency and nuclease resistance. Here, we showed that the DNA nanoparticles (DNPs) can be prepared with RCA and modified nucleotides that have side-chains appended on the nucleobase are capable of interacting with the DNA strands of the resulting RCA products. The incorporation of the modified nucleotides improved cellular uptake efficiency and nuclease resistance of the DNPs. We also demonstrated that these DNPs could be employed as carriers for the delivery of a photosensitizer into cancer cells to achieve photodynamic therapy upon irradiation at both the in vitro and in vivo levels.

## 1. Introduction

**Figure 1:**
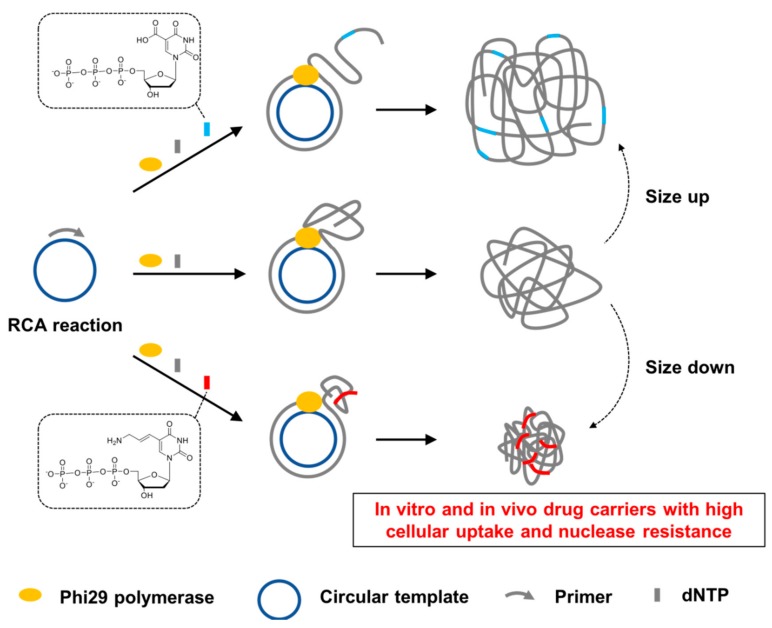
Schematic representation of the principle to control the size of rolling circle amplification (RCA) products by incorporation of chemically modified nucleotides.

Rolling circle amplification (RCA) is a DNA/RNA polymerization on a circular template using polymerases capable of strand displacement, producing long polynucleotide strands containing repetitive sequences [1,2,3]. Owing to the robust and easy protocols to synthesize polymerized DNA and RNA strands, RCA has been employed to prepare various DNA and RNA materials [4,5,6]. In particular, when shaped into nanoparticles, RCA products would have great potential to be utilized in biomedical applications, such as carriers for drug delivery, as DNA and RNA are highly biocompatible materials. Cationic lipids and polymeric amines have been widely used for nano-formulation of RCA products and have allowed for the improvement of some critical properties, such as a high cellular uptake efficiency and the nuclease resistance required for successful applications in vitro and in vivo [5,7,8,9,10]. However, as cationic lipids and polymeric amines may compromise the biocompatibility of DNA and RNA materials due to their potential cytotoxicity [11,12], the nanoparticle formation in a self-assembled manner without external additives is a more valid approach, especially when cellular and in vivo applications are considered. Previously, the size of RCA products could roughly be controlled to form nanoparticles (ca. 400 nm) by varying the concentrations of polymerases [13]. More recently, DNA nanoparticles (DNPs) with more suitable sizes for cellular uptake (ca. 50–100 nm) could be produced by adjusting the sequences of the products and by incorporating the modified nucleotides bearing cationic amine side-chains that can interact with the phosphate backbones of polynucleotide strands [14]. Although the cellular uptake efficiency could be improved by adjusting the size and the sequence of DNPs in the previous study, it has not been investigated whether the DNPs can also be used in the in vivo environment rich in nucleases. In this context, we performed RCA reactions using modified nucleotides with functional groups that can interact with the DNA backbones (Figure 1). We clearly demonstrated that the incorporation of these modified nucleotides has a strong influence on the size of DNPs and thereby positively affected both the cellular uptake efficiency and nuclease resistance of DNPs.

Photodynamic therapy (PDT) is an anticancer treatment based on apoptotic reactive oxygen species produced upon irradiation on intracellularly delivered photosensitizers [15,16,17,18,19,20]. As the conventional photosensitizers used in practice have relatively low water solubility, intracellular instability, and/or low cell permeability [21,22,23,24], various nanoparticle formulation-based delivery platforms have been proposed for enhanced in vitro and in vivo delivery of the photosensitizers to ultimately improve PDT effects [25,26,27,28,29]. To demonstrate the utility of the size-controlled DNPs showing enhanced cellular uptake and nuclease resistance, we employed DNPs as carriers for in vitro and in vivo delivery of a photosensitizer to achieve photodynamic therapy (PDT).

## 2. Results and Discussion

**Figure 2:**
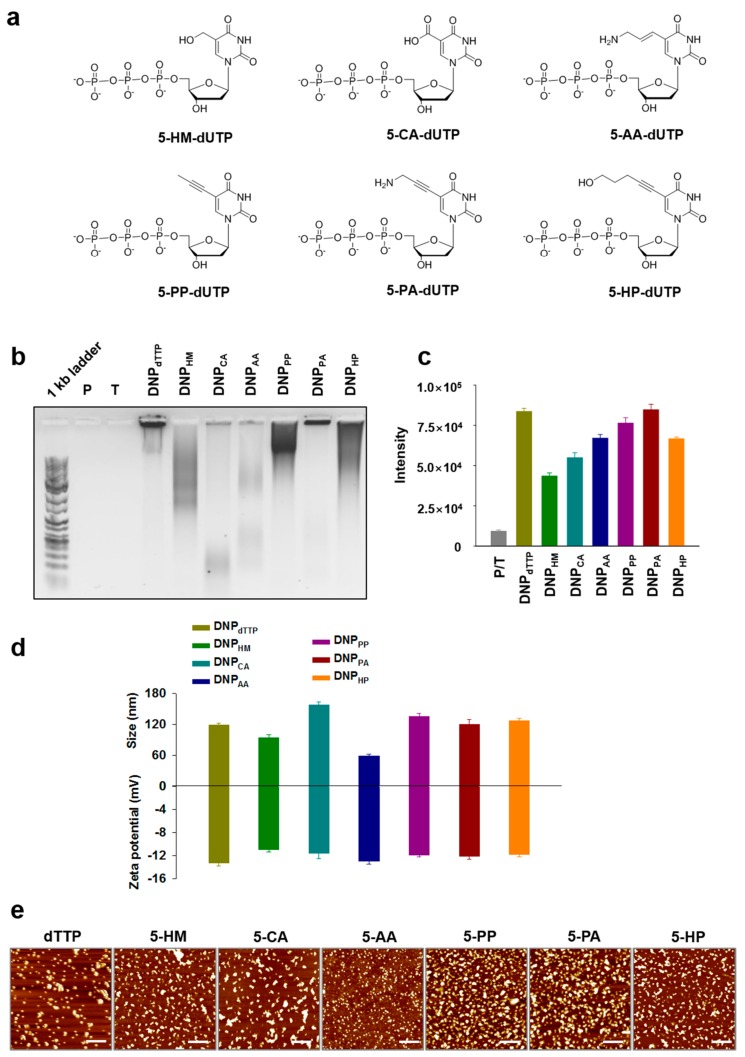
(**a**) Chemical structures of C5-modified nucleotides incorporated into DNA nanoparticles (DNPs). (**b**) Agarose gel (0.5%) analysis of RCA-produced DNPs (P: primer, T: circular template). (**c**) Fluorescence intensity of the DNPs stained with SYBR gold. (**d**) Dynamic light scattering (DLS) analysis showing hydrodynamic sizes of DNPs and zeta potential values of DNPs. (**e**) Atomic force microscopy (AFM) images of DNPs. The scale bar: 1 μm.

The circular template sequence was designed to be complementary to the AS1411 aptamer sequences following the previous study [14]. The aptamer sequences forming secondary structures are known to shape the RCA products into DNPs [14]. For an additional handle to control sizes, we incorporated chemically modified nucleotides with various functional groups such as hydroxymethyl (HM), carboxylic acid (CA), allylamine (AA), propynyl (PP), propargylamine (PA), and hydroxypentynyl (HP) at the C5 position of dUTP to fine-tune the size of the resulting DNPs via electrostatic and hydrophobic interactions (Figure 1 and Figure 2a). Agarose gel analysis of the DNPs produced by RCA reactions showed different band patterns depending on the modifications of the nucleotides, indicating that that incorporation of the modified nucleotides influenced the length and the yield of the RCA products (Figure 2b). The relative yields of the RCA reactions with C5-modified dUTPs were also estimated by measuring UV absorbance at 260 nm (Figure S1) and fluorescence intensity of the products stained with a fluorogenic DNA binding dye (SYBR gold) (Figure 2c). Compared with the RCA reaction performed using natural dTTP, the RCA reaction performed with a mixture of 5-HM-dUTP and dTTP (1:4) yielded approximately only a half of the product. When 5-PA-dUTP was used instead of 5-HM-dUTP, the yield of the RCA reaction was similar to that using dTTP.

The order of the reaction yield to form DNPs was the following: dTTP (DNP_dTTP_) ~ 5-PA-dUTP (DNP_PA_) > 5-PP-dUTP (DNP_PP_) > 5-AA-dUTP (DNP_AA_) ~ 5-HP-dUTP (DNP_HP_) > 5-CA-dUTP (DNP_CA_) > 5-HM-dUTP (DNP_HM_). Hydrodynamic sizes of DNPs were measured by dynamic light scattering (DLS) (Figure 2d). Without modified nucleotides, the size of DNP_dTTP_ increases with the reaction time, which indicates that the higher RCA yield may lead to the larger size of DNPs (Figure S2). In terms of the effect of modifications on the size of DNPs, DNP_PP_ showed a slightly increased size compared with that of DNP_dTTP_, possibly due to the steric clash of the propynyl groups during DNP packing. DNP_CA_ was larger than DNP_dTTP_, which indicates that incorporation of the modified nucleotide with a carboxylic group interferes with the compact packing of polymerized DNA strands, possibly due to the repulsive interaction between the phosphate backbone and the negatively charged carboxylic group at the given pH (7.5). When the modified nucleotides with an amine and a hydroxyl group (which have attractive interactions with the phosphate backbone) were incorporated, the sizes of the resulting DNPs (DNP_HM_ and DNP_AA_) were decreased compared with that of DNP_dTTP_, suggesting that there are possible attractive interactions with the phosphate backbone and the side-chains. However, if these functional groups are accompanied with a sterically constrained or a bulky chain as in PA and HP, the sizes of the DNPs did not decrease but were similar or slightly increased compared with that of DNP_dTTP_. The surface charges of the DNPs were similar one another, displaying −10 to −15 mV zeta potential values (Figure 2d). The variable sizes of DNPs depending on the modification of the incorporated nucleotides were also imaged under atomic force microscopy (AFM), showing a consistent pattern with the DLS data (Figure 2e). Despite the modest size distribution in DLS, the DNP particles in the AFM images were in a wider range of size distribution, because DNPs could be collapsed in different ways and be aggregated to form larger particles after dried on mica.

**Figure 3:**
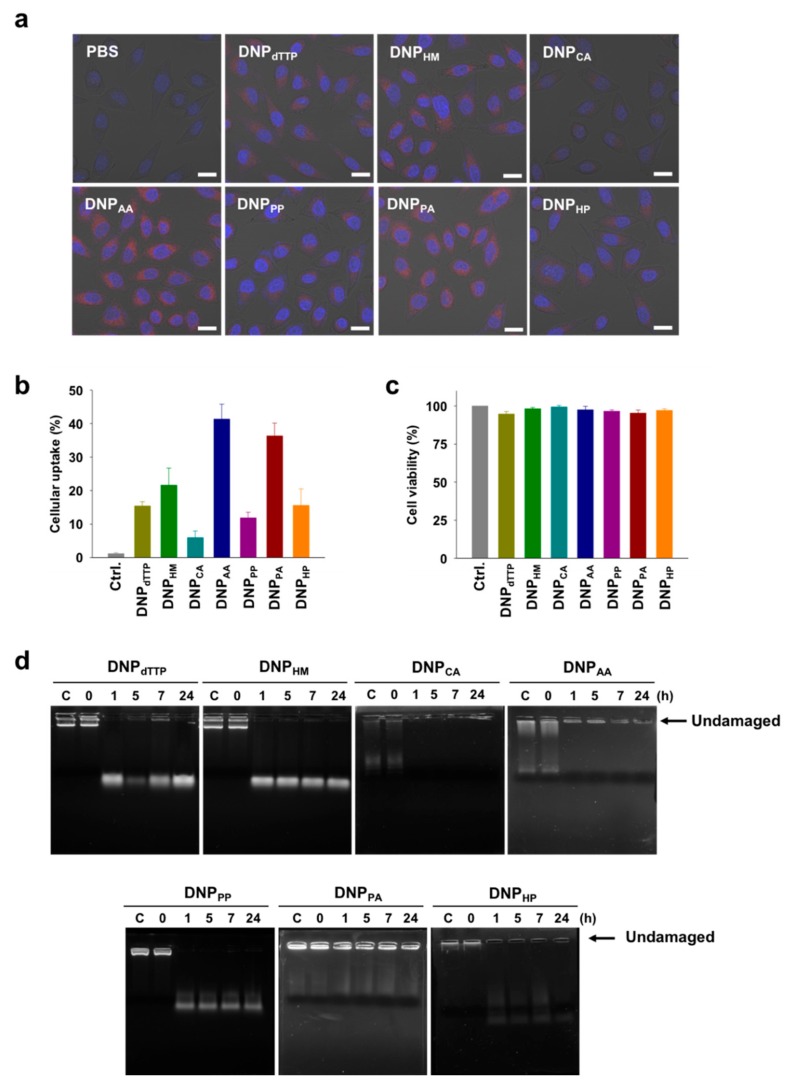
(**a**) Confocal fluorescence microscopic images of cells treated with Cy5-labeled DNPs (10 nM). Red: Cy5-DNPs, blue: nuclei. Scale bar: 25 μm. (**b**) Cellular uptake efficiency of Cy5-labeled DNPs measured by flow cytometry. (**c**) Viability of cells treated with DNPs (10 nM). (**d**) Agarose gel electrophoresis of DNPs after incubation with DNase I. The arrow indicates the position of undamaged DNPs.

Having found that various sizes of DNPs could be produced by RCA using chemically modified nucleotides, we next considered the utilization of DNPs as drug delivery carriers. In this context, we examined whether the resulting DNPs possessed the properties required to serve as drug delivery carriers, namely improved cellular uptake and nuclease resistance. For the cellular uptake test, DNPs were labeled with Cy5 by performing RCA with a Cy5-labeled primer. The uptake of DNP_dTTP_ was based on energy-dependent endocytosis, as the uptake level was significantly decreased at a low temperature. When we treated cells with DNP_dTTP_ in the presence of endocytosis inhibitors, the uptake level was also decreased, not only by the clathrin-mediated endocytosis inhibitor (chlorpromazine), but also by the non-clathrin-mediated endocytosis inhibitors (methyl-β-cyclodextrin and 5-(*N*-ethyl-*N*-isopropyl) amiloride) and the scavenger receptor-mediated endocytosis inhibitor (poly-inosinic acid) (Figure S3). This result indicates that various types of endocytosis mechanisms are involved in the uptake of DNPs. Fluorescence microscopic images of HeLa cells treated with the Cy5-labeled DNPs revealed that the cellular uptake of DNP_AA_, DNP_AP_, and DNP_HM_ is higher than that of DNP_dTTP_ (Figure 3a). The uptake efficiency was also quantitatively estimated by measuring the level of Cy5-positive HeLa cells using flow cytometry after treatment of the cells with Cy5-labeled DNPs (Figure 3b). The order of cellular uptake efficiency was DNP_AA_ > DNP_AP_ > DNP_HM_ > DNP_dTTP_ ~ DNP_HP_ > DNP_PP_ > DNP_CA_. DNPs that were obtained with amino-modified nucleotides showed relatively high cellular uptakes, whereas the DNPs modified with carboxylic acid groups displayed only a limited cellular uptake. Among DNPs modified with non-charged groups, the smaller sized one showed higher cellular uptake level. These results suggest that cellular uptake of the DNPs produced by RCA can be enhanced by decreasing the size and introducing positively charged groups into the nanoparticles. In addition, all the modified DNPs did not show any considerable cytotoxicity, illustrating that DNPs are highly biocompatible materials (Figure 3c). We also examined the nuclease resistance of the DNPs which is required particularly for their in vivo applications (Figure 3d). The DNPs treated with DNase I were analyzed by agarose gel electrophoresis. Most of the DNPs were degraded by DNase I into small DNA fragments within an hour. However, DNP_AA_ and DNP_PA_ showed significantly high nuclease resistance, with a considerable amount of undamaged DNA constructs remaining even after the treatment with DNase I for several hours. It seems that the outer layer of DNPs degraded easily, while the core constructs are stable against nucleases. This nuclease stability of the core part could be due to the serum stability of the AS1411 sequence generated in DNPs [30]. Depending on the chemical modifications embedded, the secondary structure of AS1411 could be weakened or strengthened, which resulted in differentiation of the nuclease stability of DNPs.

**Figure 4:**
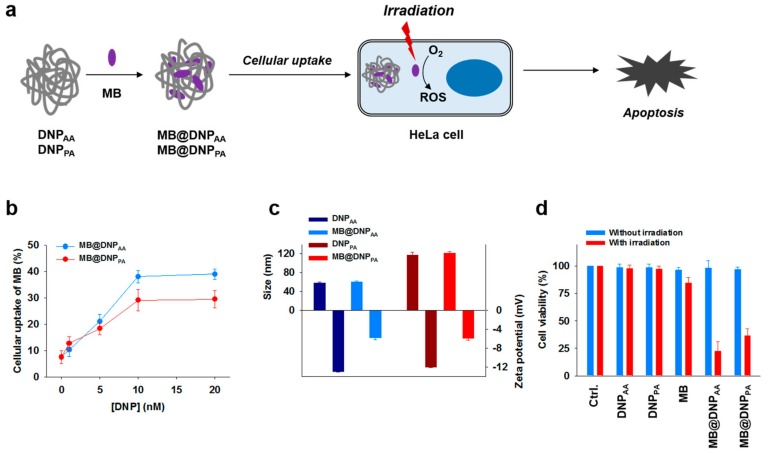
(**a**) Schematic presentation of in vitro photodynamic therapy (PDT) after the intracellular delivery of methylene blue (MB) into HeLa cells using DNP_AA_ and DNP_PA_ as carriers. (**b**) Quantitative analysis of cellular uptake of MB (1 μM) by DNPs at various concentrations using a flow cytometer. The concentrations of DNPs were determined based on the primer concentrations used for RCA. (**c**) Hydrodynamic sizes and zeta potential values of MB@DNPs compared with those of DNPs. (**d**) Viability of HeLa cells treated with MB, DNPs, and MB@DNPs before (blue) and after (red) laser irradiation. Untreated cells were used as the control (Ctrl.).

Having observed that DNP_AA_ and DNP_PA_ have considerable cellular uptake properties and enhanced nuclease resistance, we attempted to use them as carriers for the intracellular delivery of methylene blue (MB), a photosensitizer which can induce apoptotic cell damage for photodynamic therapy (PDT) upon irradiation (Figure 4a). As MB is capable of interacting with and binding to DNA [31], we determined the optimal binding ratio between MB and DNPs for drug loading by examining the cellular uptake efficiency of MB-loaded DNPs (MB@DNPs) at various DNP concentrations. At 1 µM MB, the uptake level of the photosensitizer was increased with higher concentrations of DNPs. The optimal MB@DNPs were prepared using a mixture of 1 µM MB and 10 nM DNPs because a DNP concentration higher than 10 nM did not lead to further increases in the cellular uptake of MB (Figure 4b). While the MB-loading into DNPs did not affect the size of DNPs, their zeta potential values became less negative after the MB-loading (Figure 4c). This is due to the positive charge of MB that can partially mask the negative charge of the backbone of the DNPs. Treatment of cells with MB@DNPs did not show any considerable cytotoxicity before irradiation (665 nm), whereas the significantly reduced viability of the MB@DNPs-treated cells was observed upon irradiation (Figure 4d). However, the treatment of free MB followed by irradiation could not induce the PDT effect, possibly because of the low cell permeability and the poor intracellular stability of MB [32,33,34]. In addition, DNPs without MB were not cytotoxic regardless of irradiation, suggesting that DNPs have a potential as biocompatible drug carriers.

**Figure 5:**
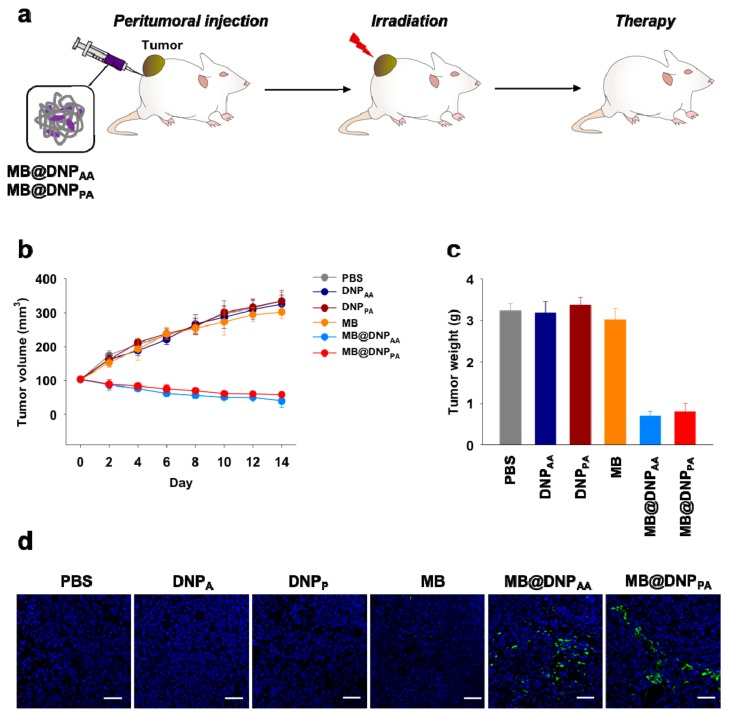
(**a**) Schematic presentation of in vivo PDT using MB@DNPs. (**b**) In vivo tumor growth profiles of mice treated with PDT using MB (orange), MB@DNP_AA_ (blue), and MB@DNP_AP_ (red). The gray circles represent untreated mice. The results indicate mean ± s.d. (*n* = 5). (**c**) Ex vivo weights of the excised tumor tissues 14 days post-treatment. (**d**) Histological images of the tumor tissues after staining with Hoechst 33458 (nuclei, blue) and fluorescein-labeled annexin V (apoptotic cells, green). Scale bar: 50 µm.

After demonstrating the feasibility of DNPs as carriers for intracellular delivery of the photosensitizer for PDT, we finally tested their in vivo potency in xenograft tumor-bearing mice. MB, MB@DNPs, or DNPs were peritumorally injected into HeLa tumor-bearing mice every two days, and the tumor was irradiated after every injection. The tumor volume of mice injected with MB@DNPs was decreased to 50% of the initial volume (Figure 5a). By contrast, the tumors of the mice injected with free MB and DNPs increased similarly to those of the control group injected with PBS (Figure 5b). The weights of the excised tumors from MB@DNP-treated mice measured after two weeks were also significantly lower than those from MB-treated and DNP-treated groups (Figure 5c).

To examine whether the anti-tumor effect was indeed due to apoptotic damage by the PDT effect, we further analyzed the sections of the tumors from the treated mice. When the sections were stained with fluorescently labeled annexin V known to bind to apoptotic cells, only the tumors from MB@DNP-treated mice showed fluorescent regions while apoptotic damage was not observed in the tumors from free MB-, DNPs-, and PBS-treated mice. These results indicate that the in vivo PDT effect can be obtained when MB was delivered by the DNPs, which can provide enhanced cellular uptake properties and high nuclease resistance.

## 3. Methods

### 3.1. C5-Modified dUTP

5-PA-dUTP, 5-PP-dUTP, 5-AA-dUTP, 5-CA-dUTP and 5-HM-dUTP were purchased from TriLink Biotechnologies (San Diego, CA, USA). 5-HP-dUTP was synthesized by application of literature protocols [35,36].

### 3.2. Preparation of the Circular Template for RCA Reactions

The circular template for RCA reactions was prepared following the previous report [14]. Briefly, 5′-phosphorylated linear template (Bioneer, Daejeon, Korea) (phosphate-CTCACCAGAGCCACCACCACCAACACCACCACCACCAAAAAAACCACCACCACCAACACCACCACCACCAAGTCCTGTC) (10 μM) was incubated with primer (15 μM, CTCTGGTGAGGACAGGACTT), 10× ligation buffer (1×) and T4 DNA ligase (400 units/μL) (New England Biolabs, Ipswich, MA, USA) at 16 °C overnight. After ligation, the circular template was treated with Exonuclease I and III (New England Biolabs), and then purified by 10% denaturing PAGE followed by ethanol precipitation.

### 3.3. RCA Reaction

The solutions containing circular templates (150 nM), natural dNTP (0.2 mM) (Promega, Madison, WI, USA), 5-modified dUTP (in a 1:4 ratio with natural dTTP), the primer (150 nM), phi29 polymerase (10 units/μL) (Lucigen, Middleton, WI, USA) and the reaction buffer (1×) of phi29 provided by the manufacturer were incubated at 30 °C for 1 h. Then, the reaction mixtures were inactivated at 65 °C for 10 min.

### 3.4. Agarose Gel Electrophoresis

Agarose gels (0.5%) (Lonza, Basel, Switzerland) were run in 0.5× TBE buffer (Biosesang, Seongnam, Korea) at 100 V for 45 min at 25 °C. After electrophoresis, the images were visualized using a fluorescence scanner (ChemiDoc, Bio-Rad, Hercules, CA, USA). RCA products were stained with SYBR gold (1×) (Molecular Probes, Eugene, OR, USA).

### 3.5. Fluorescence Intensity of SYBR Gold-Stained DNPs

DNPs produced by RCA reactions were stained with SYBR gold for 15 min at 25 °C. The fluorescence intensity of the solutions was measured using a fluorescence microplate reader (Appliskan, Thermo Fisher Scientific, Waltham, MA, USA). Excitation at 485 nm and emission at 513 nm were employed for the measurement.

### 3.6. UV Absorbance of RCA Products

After RCA reactions, RCA products were dialyzed in 0.1× PBS overnight at 25 °C by using Amicon filters (MWCO = 100 K, Millipore, Burlington, MA, USA) to remove the unreacted dNTPs. Then, UV absorbance of the products was measured at 260 nm.

### 3.7. Dynamic Light Scattering (DLS)

The hydrodynamic sizes and zeta potential of the DNPs and MB@DNPs were measured in a Zetasizer (Malvern, UK). The concentration of the samples used for the DLS analysis was 150 nM.

### 3.8. Atomic Force Microscopy (AFM)

AFM samples (10 μM) were deposited onto freshly cleaved mica (Pelco Mica sheets, Ted Pella, Redding, CA, USA). After 1 h, the mica surface was rinsed with distilled water and immediately dried using nitrogen gas. The samples were scanned in non-contact mode on a Park XE-100 (Park Systems Corp., Suwon, Korea) with PPP-NCHR tips (Park Systems Corp., Suwon, Korea). AFM data were analyzed using XEI 4.1.0 software.

### 3.9. Cellular Uptake of DNPs

Before being added to cells, the RCA products were dialyzed in 0.1× PBS overnight at 25 °C using size exclusion filters (Amicon^TM^, MWCO = 100 K, Millipore, Burlington, MA, USA). The samples were lyophilized, resuspended in distilled water and quantified based on the UV absorption at 260 nm. For microscopic analysis, HeLa cells were plated in glass-bottomed 35 mm dishes (SPL, Pocheon, Korea) with DMEM media (Welgene, Gyeongsan, Korea) containing 10% heat inactivated fetal bovine serum (Welgene, Gyeongsan, Korea) and 1% penicillin and streptomycin (Welgene, Gyeongsan, Korea). After cells (2 × 10^4^) were seeded in each dish, the dishes were incubated overnight at 37 °C in a humidified atmosphere containing 5% CO_2_. Growth medium was removed from each cell sample, and the cells were washed twice with DPBS (Welgene, Gyeongsan, Korea). DNPs (produced with the Cy5-labeled primer, 10 nM) in fresh media (250 μL) lacking serum and antibiotics was then added to a sample of cells and incubated for 6 h at 37 °C in a CO_2_ incubator. Cells then were washed twice with PBS, nuclei were stained using 3 μg/mL Hoechst 34580 (Invitrogen, Carlsbad, CA, USA), and the cells were washed with DPBS (200 μL) twice. DPBS (200 μL) was then added. Live cells were imaged using a fluorescence microscope (LSM 700, Carl Zeiss Microscopy, Oberkochen, Germany). Excitation/emission filters used for Cy5 and Hoechst 34580 were 630–650/665–705 nm, and 340–380/432–482 nm, respectively. For flow cytometry analysis, cells were seeded on 12-well culture plates (SPL, Pocheon, Korea) and cultured for 24 h at 37 °C with 5% CO_2_, and then washed twice with DPBS. They were incubated with the Cy5-labeled DNPs prepared by using a Cy5-labeled primer (10 nM) for 6 h at 37 °C with 5% CO_2_, harvested, and washed three times with DPBS. Then, 0.2 mL of trypsin EDTA was added to each sample, and the samples were incubated for 5 min at 37 °C. Then, 1 mL of media was added to each sample, and the resulting cell suspensions were transferred to 1.5 mL tubes (Axygen, Corning, NY, USA) and centrifuged for 3 min at 1500 *g*. The supernatant was removed, and the cell pellets were resuspended in 0.5 mL of DPBS. Fluorescence intensity of the cells was determined by flow cytometry (Guava, Millipore, Burlington, MA, USA). Samples of at least 10,000 cells were analyzed in triplicate.

### 3.10. Cellular Uptake Mechanism

For endocytosis inhibitor treatment, HeLa cells were seeded (5 × 10^4^ cell/well) onto 24-well plate and incubated at 37 °C, in a 5% CO_2_ incubator. After 24 h, the cells were then washed twice with PBS and then pre-incubated with various inhibitors such as chlorpromazin (CPZ, 10 μM), methyl-β-cyclodextrin (MβCD, 1 mM), ethylisopropylamiloride (EIPA, 50 μM) or poly-inosinic acid (poly-I, 50 μg/mL) in serum free DMEM for 30 min before the addition of the RCA product (DNP_dTTP_, 10 nM). After 6 h, the cells were washed twice with PBS, trypsinized, then washed with cold PBS twice, resuspended in cold PBS (500 μL), and analyzed by flow cytometry (Guava, Millipore, Burlington, MA, USA). Samples of at least 10,000 cells were analyzed in triplicate. The relative internalization level was displayed as the percentage value with mean ± standard deviation (SD), compared to the cellular uptake level in the inhibitor untreated HeLa cells as the control.

### 3.11. Cytotoxicity Analysis of DNPs

Cytotoxicity of DNPs was analyzed via the CCK-8 assay kit (Dojindo Laboratories, Kumamoto, Japan). HeLa cells were seeded in a 96-well culture plate (SPL, Pocheon, Korea) and incubated at 37 °C in a humidified atmosphere containing 5% CO_2_. When the confluency of cells was 80%, the cells were washed with DPBS. DNPs were added into the plate and incubated for 24 h at 37 °C. The plates were washed twice with DPBS and treated with 10 μL CCK-8 solution in fresh medium (100 μL) lacking serum and antibiotics. After 4 h incubation, the absorbance was measured at 450 nm using a microplate reader (SpectraMax^TM^ Plus, Molecular Devices, San Jose, CA, USA).

### 3.12. Nuclease Resistance

For the stability test, 10% DNase I (10 μL, New England Biolabs, Ipswich, MA, USA) was added to the DNA solutions (90 μL, 900 nM), and the mixtures were incubated at 37 °C. At each time point, the solutions were quenched by adding the stop solution, composed of 98% deionized formamide, 10 mM EDTA, 0.5 mg/mL bromophenol blue and xylenecyanole, and then analyzed on agarose gel pre-stained with SYBR gold. The amount of undamaged DNPs was estimated by visualization on a fluorescence scanner (Chemidoc, Bio-Rad, Hercules, CA, USA).

### 3.13. Preparation of MB@DNPs

To prepare MB@DNPs, MB (1 μM) (Sigma-Aldrich, St. Louis, MO, USA) was mixed with the DNP_AA_ and DNP_PA_ (1, 5, 10 and 20 nM) at room temperature for 1 h.

### 3.14. Cellular Uptake of MB@DNPs

HeLa cells were seeded with complete DMEM media (1 mL) onto 12-well plates and incubated at 37 °C in the CO_2_ chamber for 24 h. The media was replaced with the fresh media containing free MB (1 μM) or MB@DNPs (with 1 μM MB loaded), and then incubated at 37 °C for 6 h in the CO_2_ chamber. The cells were washed with DPBS twice, harvested and suspended in DPBS (1 mL) for flow cytometry analysis.

### 3.15. In Vitro PDT

Cells were cultured in 96-well plates and treated with MB@DNPs (prepared by mixing 1 nM DNPs and 100 nM MB) or free MB (100 nM) for 6 h under the same conditions used for the cytometric analysis. After washing with fresh media, the plate was incubated for 24 h with or without laser irradiation (665 nm and 200 mW·cm^−2^) on each well. The cell viability was then evaluated via CCK-8 assay.

### 3.16. Xenograft Tumor-Bearing Mice

The animal study was approved by the animal care and use committee of the Korea Institute of Science and Technology and all mice were handled in accordance with institutional regulations (2017-110). For tumor model preparation, mice were anaesthetized with intraperitoneal injection of zoletil-rompun mixture. Animal disease models were prepared on BALB/c nude mice (male, 5 weeks old, Orient Bio Inc., Seongnam, Korea). Tumors were established by subcutaneous inoculation of HeLa cells (1.0 × 10^7^ cells suspended in the culture medium) into the thigh of mice.

### 3.17. In Vivo PDT

After tumor growth to ca. 100 mm^3^ size, the mice were administered a peritumoral injection of MB (100 μM, 100 μL), MB@DNP_AA_, MB@DNA_PA_ (prepared by mixing 100 μM MB and 1 μM DNA, 100 μL), or PBS (100 μL). After 30 min post injection, the tumors were exposed to a laser light (665 nm) for 15 min. This PDT treatment was performed once every two days. The tumor volume was estimated by using the digital caliper before laser irradiation and calculated according to the formula (a^2^b)/2, where a and b were the respective width and length of the tumor.

### 3.18. Histological Analysis

After the last treatment, the tumors were excised from mice and fixed in 4% formaldehyde, embedded in paraffin, and cut into 5 μm sections. For fluorescent histological analysis, sections were treated with annexin V (Annexin V-FITC Apoptosis Detection Kit, Sigma-Aldrich, St. Louis, MO, USA) that can bind to apoptotic cells and Hoechst 34580 (Invitrogen, Carlsbad, CA, USA) for nuclei staining, and then analyzed by confocal microscopy (LSM700, Carl Zeiss Microscopy, Oberkochen, Germany).

## 4. Conclusions

In this study, we prepared DNPs with RCA reactions using nucleoside triphosphates bearing various modifications on the nucleobase. The size of the DNPs can be modulated and controlled by incorporating chemically modified nucleotides with various functional groups that can interact with the DNA backbone. The controlled size and the nature of the modification both influenced on the cellular uptake and nuclease resistance of DNPs, which enabled us to utilize DNPs as carriers for in vitro and in vivo drug delivery of a photosensitizer to obtain the PDT effect. Therefore, the approaches shown here to engineer DNPs produced by RCA that are suitable for in vitro and in vivo applications will provide an alternative way to produce drug delivery carriers with high biocompatibility.

## Supplementary Materials

The following are available online, Figure S1: UV absorbance of DNPs at 260 nm, Figure S2: DLS analysis showing hydrodynamic sizes of DNPdTTP produced by RCA at various reaction times, Figure S3: Relative cellular uptake of DNPdTTP into HeLa cells in the presence and absence of endocytosis inhibitors. Chlorpromazine (CPZ) is an inhibitor for the clathrin-dependent endocytosis. Methyl-β-cyclodextrin (MbCD) is an inhibitor for the caveolae-dependent endocytosis. 5-(N-ethyl-N-isopropyl) amiloride (EIPA) is an inhibitor for macropinocytosis. Poly-inosinic acid (poly-I) is an inhibitor for scavenger receptor-mediated endocytosis. The uptake levels were measured by flow cytometry. The data show the results of triplicated experiments.
