# Supplementary material for: Shaping Rolling Circle Amplification Products into DNA Nanoparticles by Incorporation of Modified Nucleotides and Their Application to In Vitro and In Vivo Delivery of a Photosensitizer
Source: Molecules. 2018 Jul 23;23(7):1833. doi: 10.3390/molecules23071833 (PMC6099487; doi:10.3390/molecules23071833)
Supplement: Supplementary file 1 [file molecules-23-01833-s001.pdf]

Supporting Information

Shaping rolling circle amplification products into DNA nanoparticles by incorporation of modified nucleotides and their application to *in vitro* and *in vivo* delivery of a photosensitizer

Kyoung-Ran Kim[a], Pascal Röthlisberger[c], Seong Jae Kang[a], Kihwan Nam[d], Sangyoup Lee[d], Marcel Hollenstein[c], and Dae-Ro Ahn[a,b,*]

a. Center for Theragnosis, Biomedical Research Institute, Korea Institute of Science and Technology (KIST), Hwarangno 14-gil 5, Seongbuk-gu, Seoul 02792, Korea

b. Division of Biomedical Science and Technology, KIST School, Korea University of Science and Technology (UST), Hwarangno 14-gil 5, Seongbuk-gu, Seoul 02792, Korea

c. Laboratory for Bioorganic Chemistry of Nucleic Acids, Department of Structural Biology and Chemistry, Institut Pasteur, CNRS UMR3523, 28, rue du Docteur Roux, 75724 Paris Cedex 15, France

d. Center for Bionics, Biomedical Research Institute, Korea Institute of Science and Technology (KIST), Hwarangno 14-gil 5, Seongbuk-gu, Seoul 02792, Korea

*To whom correspondence should be addressed. E-mail: drahn@kist.re.kr

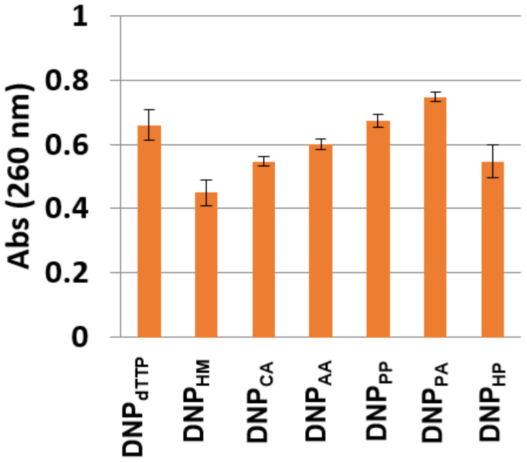

Figure S1. UV absorbance of DNPs at 260 nm

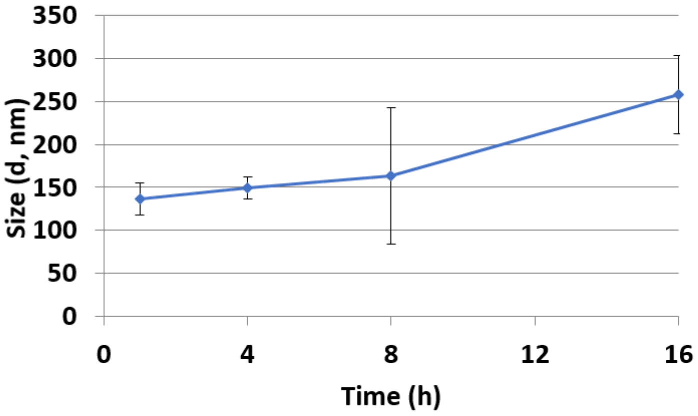

Figure S2. DLS analysis showing hydrodynamic sizes of DNP_{dTTP} produced by RCA at various reaction times.

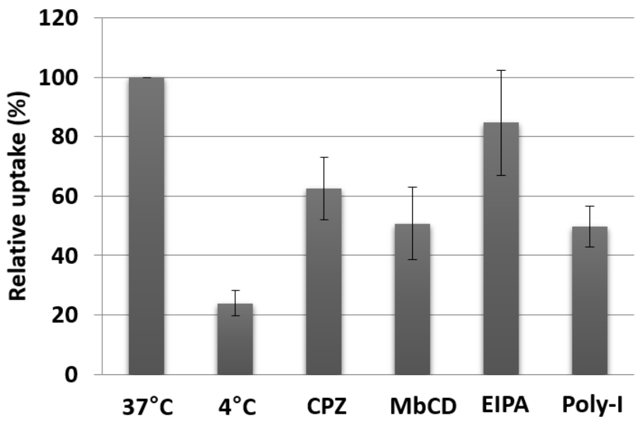

Figure S3. Relative cellular uptake of DNP_{dTTP} into HeLa cells in the presence and absence of endocytosis inhibitors. Chlorpromazine (CPZ) is an inhibitor for the clathrin-dependent endocytosis. Methyl-β-cyclodextrin (MbCD) is an inhibitor for the caveolae-dependent endocytosis. 5-(*N*-ethyl-*N*-isopropyl) amiloride (EIPA) is an inhibitor for macropinocytosis. Poly-inosinic acid (poly-I) is an inhibitor for scavenger receptor-mediated endocytosis. The uptake levels were measured by flow cytometry. The data show the results of triplicated experiments.
